# Initial report of *Panstrongylus rufotuberculatus* (Champion, 1899) Triatominae (Hemiptera: Reduviidae) in Roraima, western Amazon state, Brazil

**DOI:** 10.1590/0037-8682-0113-2022

**Published:** 2022-10-24

**Authors:** Éder dos Santos Souza, Dionatas Ulises de Oliveira Meneguetti, Cleber Galvão, Vinicius Fernandes de Paiva, Izabele de Souza Guimarães, Ágata Cristian Lima da Silva, Gabriela Maciel Alencar, Maria das Graças Vale Barbosa Guerra

**Affiliations:** 1 Universidade do Estado do Amazonas, Fundação de Medicina Tropical Doutor Heitor Vieira Dourado, Centro de Entomologia, Programa de Pós-graduação em Medicina Tropical, Manaus, AM, Brasil.; 2 Universidade Federal do Acre, Colégio de Aplicação, Programa de Pós-Graduação em Ciências da Saúde na Amazônia Ocidental, Rio Branco, AC, Brasil.; 3 Fundação Oswaldo Cruz, Instituto Oswaldo Cruz, Laboratório Nacional e Internacional de Referência em Taxonomia de Triatomíneos, Rio de Janeiro, RJ, Brasil.; 4 Universidade Estadual de Campinas, Instituto de Biologia, Departamento de Biologia Animal, Campinas, SP, Brasil.

**Keywords:** Chagas disease, Disease vectors, Reduviidae, Triatominae

## Abstract

**Background:**

This study is the first report of the species *Panstrongylus rufotuberculatus* in Roraima, a state in northern Brazil.

**Methods:**

We collected specimens from a residence in the municipality of Rorainópolis.

**Results:**

Our findings confirmed the occurrence of this species in Roraima, increasing the number of registered species from six to seven.

**Conclusions:**

Future studies are required to further investigate and expand our knowledge of the occurrence of this species and its epidemiological importance for this state.

Chagas disease, or American trypanosomiasis, is a neglected disease caused by the protozoan *Trypanosoma cruzi*. This disease is transmitted by blood-sucking insects of the Reduviidae family and Triatominae subfamily^1^.

Worldwide, 157 species of triatomines have been described, with at least 67 occurring in Brazil and 20 in the Brazilian Amazon^2^. In the state of Roraima, six species have been described (distributed in three genera): *Panstrongylus geniculatus* (Latreille, 1811), *Triatoma maculata* (Erichson, 1848), *Rhodnius pictipes* (Stål, 1972), *Rhodnius robustus* (Larrousse, 1927), *Rhodnius montenegrensis* (Rosa et al., 2012), and *Eratyrus mucronatus* (Stål, 1859)^2^ ^-^ ^4^. However, based on comparisons with other states in the western Amazon, the number of triatomines has likely been underestimated since eight to eleven species are known in the neighboring states of Acre, Amazonas, and Rondônia^5^ ^-^ ^7^.

The current study describes the first report of *P. rufotuberculatus* in Roraima, a state in the western Brazilian Amazon. 

A specimen of *P. rufotuberculatus* was collected from inside a residence in the municipality of Rorainópolis (latitude: 9°29’971”S, longitude: 60°40’8647”W). The specimen was found in a spider's web, from which it was removed and sent to the Department of Entomology, Tropical Medicine Hospital *Heitor Vieira Dourado*. The morphological characteristics described by Lent and Wygodzinsky and Galvão^8^ ^,^ ^9^ were used to identify the specimen as *P. rufotuberculatus*. 

The collected specimen was reasonably preserved although dry, and for this reason, we did not search for trypanosomatids to avoid damaging the specimen. *P. rufotuberculatus* (Figure 1) has the following characteristics: golden bristles on the dorsal surface of the body; reddish tubercles on the anterior lobe of the pronotum; a scutellum process that is short, rounded, conical, or truncated at the tip; connective segments with dark spots in the center; and light green forewings^8^ ^,^ ^10^. 

**FIGURE 1:**
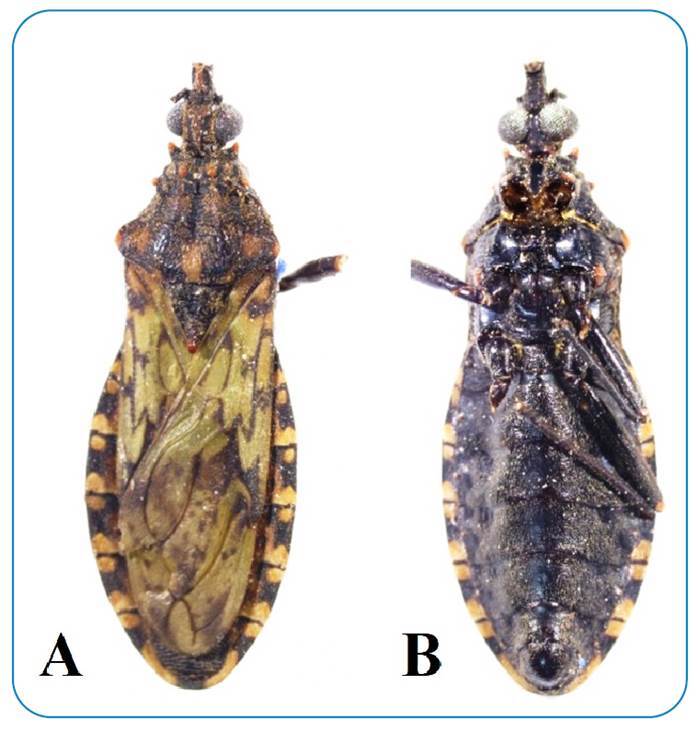
Male *P. rufotuberculatus*. **(A)** Dorsal and **(B)** ventral views.

This reported occurrence of *P. rufotuberculatus* in Roraima expands the geographic distribution of the species in Brazil to six states: Acre, Amazonas, Mato Grosso, Pará, Rondônia^5^ ^,^ ^6^ and Roraima, all of which form part of the Brazilian Legal Amazon (Figure 2). This species exists in other countries such as: Argentina, Bolivia, Colombia, Costa Rica, Ecuador, French Guiana, Mexico, Panama, Peru, Suriname, and Venezuela^7^ ^,^ ^9^ (Figure 2).

**FIGURE 2: Record:**
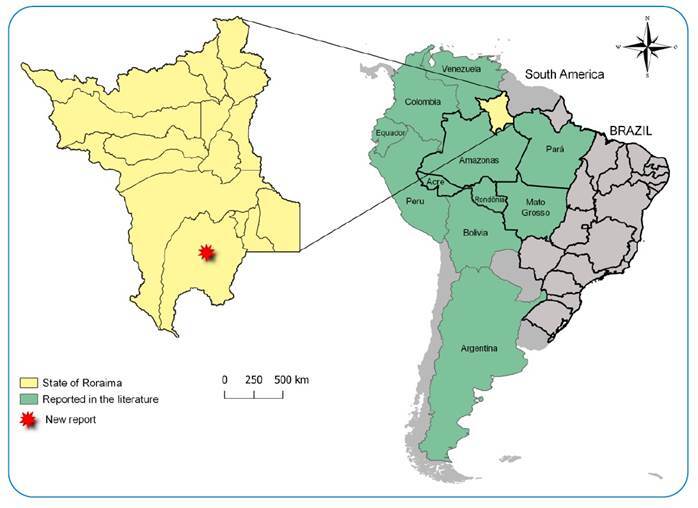
of *P. rufotuberculatus*reported in South America.

Although *P. rufotuberculatus* has not been considered a species of great epidemiological importance, it has been found in dwellings (i.e., invasion and domiciliation and peridomiciliary environments)^7^. This characteristic creates the need for a public health alert, since this species is known to be naturally infected with *Trypanosoma cruzi*, as observed in different South American countries^11^ ^-^ ^13^, and occurring from Argentina to Venezuela^14^ ^,^ ^15^.

The reported occurrence of this triatomine in Roraima increases the number of species described in the state from six to seven, demonstrating the need for future studies to better understand this occurrence and its epidemiological importance for this state. This knowledge may support the development of prophylactic measures for Chagas disease throughout the state.
